# Cyberbullying, mental health, and substance use experimentation among early adolescents: a prospective cohort study

**DOI:** 10.1016/j.lana.2025.101002

**Published:** 2025-05-20

**Authors:** Jason M. Nagata, Joan Shim, Priyadharshini Balasubramanian, Alicia W. Leong, Zacariah Smith-Russack, Iris Y. Shao, Abubakr A.A. Al-Shoaibi, Christiane K. Helmer, Kyle T. Ganson, Alexander Testa, Orsolya Kiss, Jinbo He, Allison K. Groves, Sarah Baird, Fiona C. Baker

**Affiliations:** a Division of Adolescent and Young Adult Medicine, Department of Pediatrics, University of California, San Francisco, CA, USA; b Factor-Inwentash Faculty of Social Work, University of Toronto, Toronto, ON, Canada; c Department of Management, Policy and Community Health, University of Texas Health Science Center at Houston, Houston, TX, USA; d Center for Health Sciences, SRI International, Menlo Park, CA, USA; e School of Humanities and Social Science, The Chinese University of Hong Kong, Longgang District, Shenzhen, China; f Department of Community Health and Prevention, Drexel University Dornsife School of Public Health, Philadelphia, PA, USA; g Department of Global Health, Milken Institute School of Public Health, George Washington University, Washington, DC, USA; h School of Physiology, University of the Witwatersrand, Braamfontein, Johannesburg, South Africa

**Keywords:** Cyberbullying, Victimization, Social media, Mental health, Suicide, Substance use, Adolescents

## Abstract

**Background:**

Although cyberbullying has been linked with adverse health outcomes, most prior studies have been cross-sectional, and there are limited large-scale, prospective analyses examining cyberbullying and mental health and substance use outcomes in early adolescents. Therefore, the aim of this study was to determine prospective associations between cyberbullying, mental health, and substance use experimentation one year later in a US national cohort of early adolescents (11–12 years old).

**Methods:**

We analyzed prospective cohort data from the Adolescent Brain Cognitive Development (ABCD) Study (Year 2, N = 9799). Linear and logistic regression analyses were used to determine associations between cyberbullying victimization (exposure variable, Year 2) and mental health (depressive, anxiety, attention, somatic, oppositional defiant, conduct problems, and suicidal behaviours), and substance (alcohol, nicotine, cannabis) use experimentation outcomes (Year 3), adjusting for sociodemographic variables and mental health outcomes, suicidal behaviours, or reported substance use experimentation at Year 2.

**Findings:**

The total analysed sample comprised 9799 who were 48.4% female and racially/ethnically diverse (45.1% non-White). 8.7% reported lifetime cyberbullying victimization. Cyberbullying victimization was prospectively associated with higher depressive (β = 0.61, 95% CI 0.02–1.19), somatic (β = 1.00, 95% CI 0.42–1.57), and attention problems (β = 0.52, 95% CI 0.03–1.00), as well as suicidal behaviors (adjusted odds ratio [AOR] 2.62, 95% CI 1.73–3.98) one year later. Cyberbullying victimization was prospectively associated with higher odds of alcohol (AOR 1.98, 95% CI 1.53–2.57), nicotine (AOR 3.37, 95% 2.16–5.26), and cannabis (AOR 4.65, 95% 2.46–8.77) experimentation one year later. While cyberbullying victimization was associated with anxiety, oppositional defiant, and conduct problems in the unadjusted model, this was no longer significant after adjusting for covariates.

**Interpretation:**

Given associations with poor mental health and substance use in early adolescents, it is important to develop interventions to prevent and reduce cyberbullying. Pediatricians, parents, and educators can provide mental health support for early adolescent victims of cyberbullying.

**Funding:**

This research was supported by the 10.13039/100000865Bill and Melinda Gates Foundation (INV-048897). J.M.N. was funded by the 10.13039/100000002National Institutes of Health (K08HL159350 and R01MH135492) and the 10.13039/100000862Doris Duke Charitable Foundation (2022056).

**Research in context**

**Evidence before this study**

We searched PubMed and Google Scholar for studies about cyberbullying and mental health outcomes in adolescents, published in any language from any date up to January 11, 2024, using the following search terms: “cyberbullying,” “mental health,” “adolescen∗,” “child∗,” “substance use,” “anxiety,” “depression,” “internalizing symptoms,” “externalizing symptoms,” “attention problems,” “attention-deficit/hyperactivity disorder,” “oppositional defiant disorder,” “conduct problems,” “somatic symptoms,” “physical symptoms,” “self-harm,” and “suicid∗.” We supplemented this search with a further review of references in identified articles and our own knowledge of the existing literature. Additionally, in our review of the literature, we focused on identifying large-scale analyses which examined the prospective associations between cyberbullying involvement (victimization and/or perpetration) and mental health and substance use outcomes in early adolescents. We also included studies in the revision stage between January 11, 2024, and September 30, 2024. Our search identified several systematic reviews and meta-analyses demonstrating associations between cyberbullying involvement and adverse mental health outcomes (e.g., anxiety, depression, somatic/physical symptoms, self-harm, suicidality, and substance use). However, most systematic reviews and meta-analyses included only cross-sectional data or consolidated effect sizes from cross-sectional and longitudinal data, preventing the effective identification, characterization, and quantification of temporal relations between cyberbullying and mental health issues. Studies that investigated longitudinal links between cyberbullying and mental health outcomes among adolescents were restricted to small sample sizes and limited sociodemographic diversity. Few of these studies reported or adjusted for participants’ sociodemographic characteristics (e.g., race/ethnicity, household income, caregiver education, geographic location), obscuring the generalizability of findings. Thus, there are few existing large-scale, demographically diverse, and prospective analyses of cyberbullying involvement and mental health and substance use outcomes in early adolescents.

**Added value of this study**

In a demographically diverse national cohort of early adolescents in the US, cyberbullying victimization was prospectively associated with higher depressive, somatic, and attention-deficit/hyperactivity symptoms, as well as suicidal behaviors one year later. Cyberbullying victimization and perpetration were prospectively associated with higher odds of alcohol, nicotine, and cannabis experimentation one year later.

**Implications of all the available evidence**

Because of cyberbullying's negative health effects, it is important to develop interventions to reduce cyberbullying. Pediatricians, parents, and educators can provide mental health support for early adolescent victims of cyberbullying.

## Introduction

With children and adolescents’ increasing exposure and access to electronic devices^1^^,^^2^ and the increasing popularity of social media and other digital media platforms,^3^^,^^4^ cyberbullying has become a growing public health concern affecting children and adolescents globally.^5^, ^6^, ^7^ Cyberbullying, defined as the “willful and repeated harm inflicted [by a perpetrator to a victim] through the use of computers, cell phones, and other electronic devices,”^8^^,^^9^ may be more insidious and malicious than traditional bullying because cyberbullying can be perpetrated anonymously, occur outside of school hours, reach a wider online audience, and remain more permanently preserved online.^10^, ^11^, ^12^, ^13^, ^14^ These distinctions between cyberbullying and traditional bullying make it especially difficult for caregivers, educators, and other adult figures to act against it effectively.^11^^,^^12^ While the true prevalence of cyberbullying is challenging to estimate, existing data suggest that as many as half of adolescents with online access may be exposed to cyberbullying victimization, but estimates of cyberbullying victimization prevalence vary widely, ranging from 14 to 58% globally^7^ and from 3 to 72% in the US.^15^

Especially for adolescents (10–19 years), cyberbullying has been linked in several research papers to poor mental health. These data have been synthesized in systematic reviews and meta-analyses, showing associations between cyberbullying and adverse mental health outcomes such as anxiety, depression, somatic/physical symptoms, self-harm, suicidality, and substance use among cyberbullying victims.^16^, ^17^, ^18^, ^19^, ^20^, ^21^, ^22^, ^23^, ^24^, ^25^, ^26^, ^27^, ^28^, ^29^, ^30^ However, a significant limitation of the analytical approaches of most existing systematic reviews and meta-analyses is a predominant focus on cross-sectional findings or consolidation of effect sizes from cross-sectional and longitudinal studies.^24^ The cross-sectional design utilized in the majority of these studies limits the ability to establish temporal relationships between cyberbullying victimization as an exposure and subsequent outcomes. Thus, longitudinal studies are needed to examine correlations over time and clarify whether particular factors are antecedents or outcomes of cyberbullying victimization^6^^,^^7^^,^^23^ and whether the correlations found between cyberbullying victimization and other mental health problems are spurious and/or driven by other variables.^31^

Furthermore, existing studies that examine longitudinal links between cyberbullying victimization and mental health problems in a large, diverse cohort of adolescents are scarce. A recent meta-analysis of 34 longitudinal studies from 16 countries, designated by the World Bank as high-income, found cyberbullying victimization to be a significant predictor of lower levels of academic achievement, life satisfaction, self-esteem, and higher levels of aggression, anxiety, delinquent behaviours, depression, emotional problems, loneliness, risky sexual behaviours, self-harm, somatic symptoms, stress, substance use, and suicidal thoughts among children and adolescents.^24^ The largest study included in this meta-analysis, which examined the longitudinal impacts of cyberbullying victimization, was conducted among 3181 secondary school students living in Rotterdam and the surrounding region in the Netherlands.^32^ The meta-analysis could not account for participants’ sociodemographic factors (e.g., race/ethnicity, household income, caregiver education, and geographic location), as this information was not investigated or provided in many of the included studies, thus limiting the applicability of study findings and evidence-based recommendations.^33^^,^^34^

A more detailed investigation of the associations between cyberbullying victimization and clinically relevant categories of adolescent psychopathology is also needed to provide more targeted recommendations and strategies.^5^^,^^16^ Early adolescence is a critical period of development marked by increased peer influence and decreased caregiver involvement and oversight.^35^^,^^36^ Given that cyberbullying has been shown to increase in prevalence from late childhood to early adolescence,^37^^,^^38^ identifying the potential consequences of cyberbullying victimization during early adolescence will help inform the development of effective prevention and intervention strategies. In particular, examining cyberbullying involvement as a potential risk factor for substance use experimentation during the early adolescence developmental period could inform therapeutic targets for early, efficacious substance use disorder prevention, given growing attention to the relationship between cyberbullying and substance use,^39^ and evidence indicating that substance use experimentation before age 14 increases the risk of lifelong substance use disorder.^40^

To address gaps and limitations in existing studies, we used data from the Adolescent Brain Cognitive Development (ABCD) Study to examine the prospective relationships between cyberbullying victimization and constructs of mental health outcomes, suicidal behaviours, and substance use experimentation (alcohol, nicotine, and cannabis) one year later in a demographically diverse national cohort of early adolescents in the US. As a secondary aim, we examined the prospective associations between cyberbullying perpetration and the same mental health and substance use experimentation outcomes. Participants in the current analysis were mostly 11–12 years old and were followed for one year. We hypothesized that cyberbullying victimization would be prospectively associated with poorer mental health symptoms, suicidal behaviours, and substance use experimentation at one-year follow-up.

## Methods

We conducted prospective analyses of data from year-2 and year-3 follow-ups of the ABCD Study, a prospective cohort study. The ABCD Study is currently the most comprehensive ongoing research focused on health, brain function, and cognitive growth during adolescence. Initially, the research involved 11,875 children aged 9–10 years from 21 locations in the US, surveyed between 2016 and 2018. Comprehensive information about the ABCD Study's sample, methodology, and metrics have been described previously.^41^ The sample size was calculated to ensure sufficient power to detect medium to small effects over the study's duration, accounting for 10% attrition.^41^ During the second year of assessment (2018–2020), the majority of participants were aged 11–12 years. Participants with incomplete data on substance use (n = 1584), cyberbullying inquiries (n = 485), and sociodemographics (n = 7) were excluded in the final analysis, encompassing 9799 adolescents for all our analysis (Supplementary Figure S1). For a breakdown comparing those included and those excluded, refer to Supplementary Table S1).

### Ethical approval

Approval for the study was granted by the centralized institutional review board at the University of California, San Diego (160,091). Participation in the study was contingent upon the adolescent participants giving written assent, while their parents or guardians provided written consent.

### Measures and study variables

#### Exposures

##### Cyberbullying victimization and perpetration

Adolescent participants self-reported lifetime cyberbullying victimization based on the Cyberbullying Scale.^42^^,^^43^ Cyberbullying victimization was assessed with the question, “Have you ever been cyberbullied, where someone was trying on purpose to harm you or be mean to you online, in texts, or group texts, or on social media (like Instagram or Snapchat)?” Participants were asked, “Have you ever cyberbullied someone, where you purposefully tried to harm another person or be mean to them online, in texts or group texts, or on social media (like Instagram or Snapchat)?” to assess cyberbullying perpetration. Response options were “Yes” or “No” for both items. Validation information or psychometric properties are not available for these single-item measures.

#### Outcomes

##### Mental health

###### Child Behavior Checklist (CBCL)

The CBCL is a screening instrument composed of 112 queries for parents or guardians to reflect on various psychological symptoms and behavioural challenges in children between the ages of 4 and 18.^44^^,^^45^ The CBCL provides a dimensional assessment of child psychopathology and includes the Diagnostic and Statistical Manual of Mental Disorders, Fifth Edition (DSM-5)-oriented scales (affective/depressive, anxiety, attention-deficit/hyperactivity (ADHD), somatic, oppositional defiant (ODD), and conduct problems).^46^ The CBCL's DSM-oriented scales can be reliably used in clinical settings for screening for psychopathology and enhancing diagnostic assessment based on the DSM classification system.^47^^,^^48^ Caregivers rated statements related to their child's recent behaviour on a scale ranging from 0 (doesn't apply) to 2 (frequently applies) considering the past half-year. Using the CBCL scoring rubric, T scores were calculated.^44^

###### Kiddie Schedule for Affective Disorders (KSADS-5)—Suicidal behaviors

The Kiddie Schedule for Affective Disorders and Schizophrenia-Version 5 (KSADS-5) uses DSM-5 criteria to assess mental health disorders and suicidal behaviours.^49^, ^50^, ^51^ Its computerized version^52^ has demonstrated high reliability and validity in assessing suicidal ideation and suicide attempts in the ABCD Study.^43^ Adolescent participants who reported passive suicidal ideation, nonspeciﬁc active suicidal ideation, active suicidal ideation with a plan/method/preparation/intent, or suicide attempt were coded as having suicidal behaviours.

##### Substance use experimentation

Adolescent participants completed the ABCD Youth Substance Use Introduction and Patterns survey.^53^ Information regarding the patterns of use for alcohol, nicotine products, and cannabis were collected. Participants' experimentation (low levels of use) with any type of substance was defined as participants having tried a sip of alcohol, a puff of nicotine products, or a puff of cannabis.^54^ Participants were categorized as either “substance experimentation present” or “substance experimentation absent” for each of these substances. Additionally, an initiation of experimentation measure was included to summarize participants’ new alcohol, nicotine, and cannabis experimentation at Year 3 of the ABCD Study. This measure indicated whether a participant had experimented, meaning tried a sip or puff for the first time at Year 3 while excluding those with prior use in Year 2, any or a combination of the substances mentioned above.

#### Covariates

Sex assigned at birth (female or male), age of the participant, race/ethnicity (White, Latino, Black, Asian, Native American, other), sexual orientation, household income, and parental education status were self-reported by the participants and/or their caregivers. All covariates were collected at baseline (Year 0), except for age and sexual orientation, which were collected in Year 2. The study site and the respective mental health or substance use experimentation measure at Year 2 were also included as covariates (e.g., depressive problems at Year 2 were included as a covariate for the analysis with depressive problems at Year 3 as an outcome). Total recreational screen time was assessed through the ABCD Youth Screen Time Survey at Year 2, where participants self-reported their average daily screen time usage on weekdays and weekends for eight screen modalities (watching TV shows or movies, watching videos, playing single-player, playing multi-player games, texting, video chatting, social media, and internet). A weighted sum for weekdays and weekends was used to calculate the daily average of hours spent on each screen modality. Total recreational screen time was calculated by taking the sum of the eight screen modalities.

### Statistical analysis

Data analyses were performed in 2023 using Stata 18 (StataCorp, College Station, TX). Descriptive statistics were calculated by measuring the mean, standard deviation, and percentages of each variable. Multiple logistic regression models were conducted to estimate the associations between the exposure (cyberbullying victimization at Year 2) and the binary outcomes (suicidal behaviours and substance use experimentation at Year 3), adjusting for covariates and suicidal behaviour or substance use experimentation at Year 2. Multiple linear regression models were conducted to estimate the association between the aforementioned exposures at Year 2 and the continuous outcomes (CBCL measures at Year 3), adjusting for covariates and the respective Year 2 CBCL measures. We ran a sensitivity analysis stratifying results by sex. We ran another sensitivity analysis with total recreational screen time as an additional covariate. To identify new substance use experimentation, we excluded participants with any history of substance use experimentation at Year 2 in a sensitivity analysis with substance use experimentation outcomes. In our secondary analysis, we analyzed cyberbullying perpetration as the outcome in the multiple logistic and linear models. Sample weights based on the American Community Survey of the US Census were applied.^55^ We used Cohen^56^ and Rosenthal's^57^ guidelines to interpret effect sizes: small (Cohen's d ≤0.2, odds ratio 1–1.5) medium (Cohen's d ∼ 0.5, odds ratio 1–2.5) and large (Cohen's d ≥0.8, odds ratio 1–4) effect sizes.

### Role of the funding source

The funding sources had no role in the study design; in the collection, analysis, and interpretation of data; in the writing of the report; or in the decision to submit the paper for publication.

## Results

Table 1 describes the sociodemographic characteristics of 9799 early adolescents included in this study. Nearly half (48.4%) of the participants were female and 45.1% were self-reported non-White. A majority of the participants (87.6%) were heterosexual. Nearly a tenth (9.4%) of early adolescents reported ever having been a victim of cyberbullying, and 1.0% reported perpetrating cyberbullying.

**Table 1:** Sociodemographic characteristics of Adolescent Brain Cognitive Development (ABCD) Study participants (N = 9799).

Sociodemographic characteristics	Mean (SD)/%
Age (years), year 2	12.02 (0.66)
Sex (%)	
Female	48.4%
Male	51.6%
Race/ethnicity (%), year 0	
White	54.9%
Latino/Hispanic	19.6%
Black	15.4%
Asian	5.5%
Native American	3.2%
Other	1.4%
Household income (%), year 0	
$24,999 or less	15.8%
$25,000–$49,999	19.9%
$50,000–$74,999	18.0%
$75,000–$99,999	14.2%
$100,000–$199,999	24.3%
$200,000 and greater	7.8%
Parent's highest education (%), year 0	
College education or more	82.5%
High school education or less	17.5%
Lifetime cyberbullying victimization (%), year 2	9.4%
Lifetime cyberbullying perpetration (%), year 2	1.0%
CBCL DSM-Oriented Scales (t-scores), year 3	
Depressive problems	54.20 (6.28)
Anxiety problems	53.69 (6.22)
Somatic problems	54.74 (6.21)
Attention/deficit problems	53.41 (5.48)
Oppositional defiant problems	53.15 (5.07)
Conduct problems	52.34 (4.67)
Kiddie Schedule for Affective Disorders (KSADS), year 3	
Suicidal behaviors (%), year 3	2.8%
Substance use experimentation (%), year 3	
Alcohol	11.1%
Nicotine	2.4%
Tobacco	1.0%
Any substance use	12.3%

ABCD Study sample weights were applied based on the American Community Survey from the US Census. CBCL, Child Behavior Checklist.

**Fig. 1:**
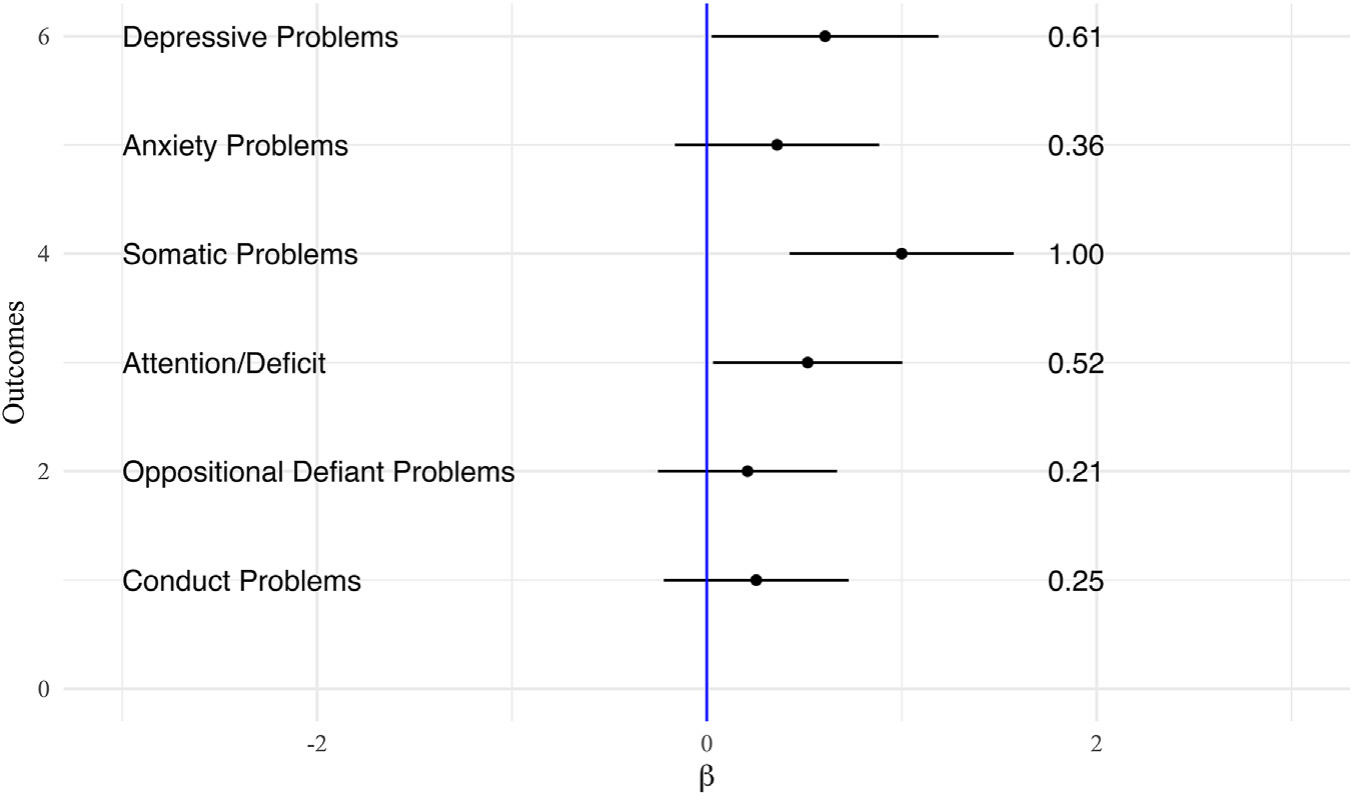
Prospective associations between cyberbullying victimization and continuous mental health outcomes one year later in the Adolescent Brain Cognitive Development (ABCD) Study (N = 9799). Forest plot of linear regression coefficients and 95% confidence intervals for associations with cyberbullying victimization (Year 2) and continuous mental health outcomes (t-scores, Year 3), adjusted for age, sex, race/ethnicity, household income, parent education, study site, and the respective mental health measure at Year 2.

**Fig. 2:**
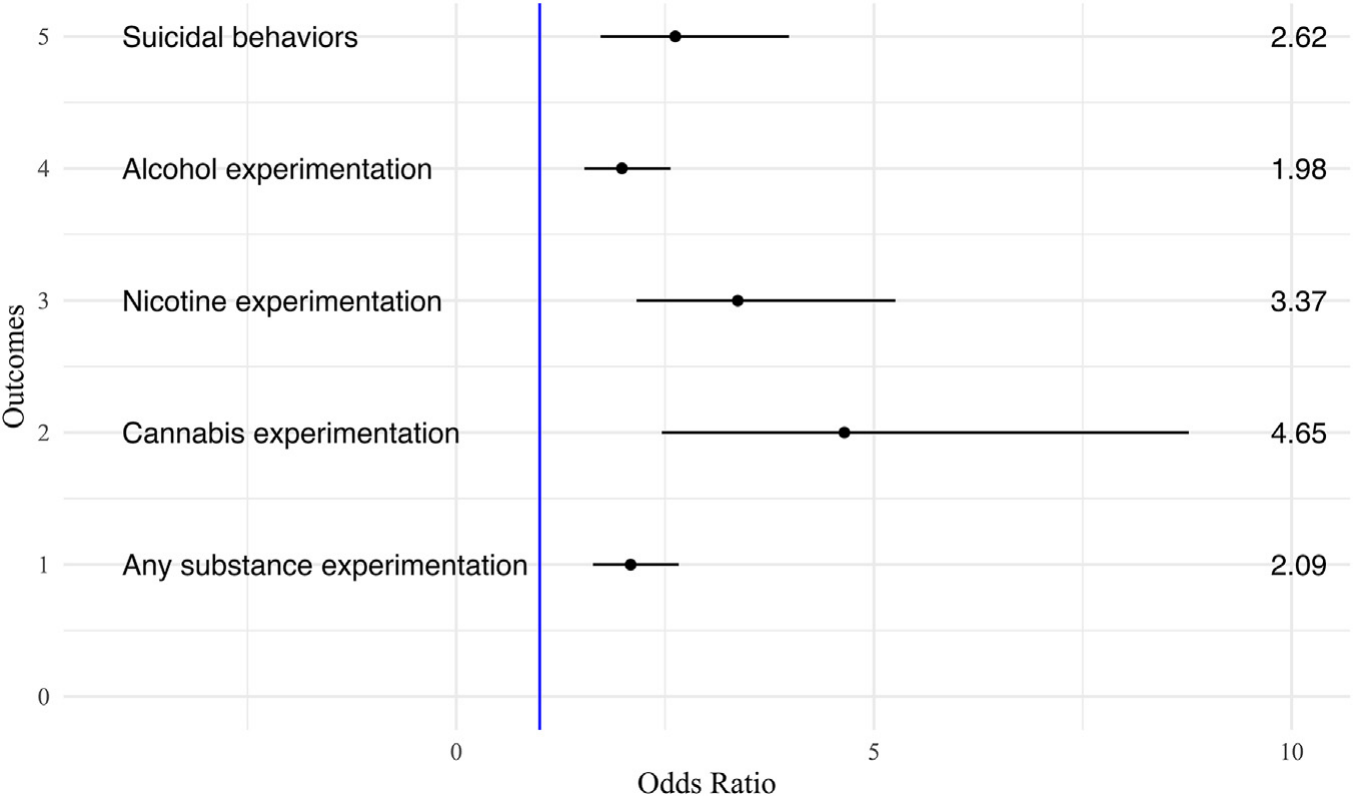
Prospective associations between cyberbullying victimization and binary suicidal behaviours and substance use experimentation outcomes one year later in the Adolescent Brain Cognitive Development (ABCD) Study (N = 9799). Forest plot of adjusted odds ratios and 95% confidence intervals from logistic regression models for associations with cyberbullying victimization (Year 2) and binary suicidal behaviours and substance use experimentation outcomes (Year 3), adjusted for age, sex, race/ethnicity, household income, parent education, study site, and the respective suicidal behaviour or mental health outcome at Year 2.

Table 2 shows associations between cyberbullying victimization and mental health and substance use experimentation outcomes. In linear regression models adjusting for covariates (Fig. 1), cyberbullying victimization was prospectively associated with increased depressive, somatic, and attention problems one year later, with small effect sizes. Particularly, those who reported cyberbullying victimization had a 0.61 (95% CI 0.02–1.19, Cohen's d = 0.10) increase in depressive problems t-score, 1.00 (95% CI 0.42–1.57, Cohen's d = 0.16) increase in somatic problems t-score, and 0.52 (95% CI 0.03–1.00, Cohen's d = 0.10) increase in attention problems t-score, one year later compared to those who did not report cyberbullying victimization. In logistic regression models adjusting for covariates (Fig. 2), cyberbullying victimization was moderately associated with alcohol and any substance use experimentation one year later. Cyberbullying victimization was associated with a 1.98 (95% CI 1.53–2.57) higher odds of alcohol experimentation and 2.09 (95% CI 1.64–2.66) higher odds of any substance use experimentation one year later compared to no cyberbullying victimization. Additionally, cyberbullying victimization was strongly associated with nicotine and cannabis experimentation, as well as suicidal behaviours, one year later. Specifically, cyberbullying victimization was prospectively associated with a 3.37 (95% CI 2.16–5.26) higher odds of nicotine experimentation, 4.65 (95% CI 2.46–8.77) higher odds of cannabis experimentation, and 2.62 (95% CI 1.73–3.98) higher odds of suicidal behaviours one year later compared to no cyberbullying victimization.

**Table 2:** Prospective associations between lifetime victimization of cyberbullying and outcomes in mental health and substance use experimentation in the Adolescent Brain Cognitive Development (ABCD) Study (N = 9799).

Outcomes at year 3 follow-up	Cyberbullying victimization		Cyberbullying victimization	
	Unadjusted		Adjusted	
CBCL DSM-oriented scales	β (95% CI)	p	β (95% CI)	p
Depressive problems	**1.85 (1.19, 2.51)**	**<0.001**	**0.61 (0.02, 1.19)**	**0.041**
Anxiety problems	**1.13 (0.50, 1.76)**	**<0.001**	0.36 (−0.16, 0.88)	0.178
Somatic problems	**1.53 (0.91, 2.14)**	**<0.001**	**1.00 (0.42, 1.57)**	**0.001**
Attention/deficit	**1.72 (1.11, 2.33)**	**<0.001**	**0.52 (0.03, 1.00)**	**0.037**
Oppositional defiant problems	**1.24 (0.68, 1.80)**	**<0.001**	0.21 (−0.25, 0.67)	0.373
Conduct problems	**1.40 (0.81, 2.00)**	**<0.001**	0.25 (−0.22, 0.73)	0.295
Kiddie Schedule for Affective Disorders	AOR (95% CI)	p	AOR (95% CI)	p
Suicidal behaviors	**3.14 (2.20, 4.48)**	**<0.001**	**2.62 (1.73, 3.98)**	**<0.001**
Substance use experimentation	AOR (95% CI)	p	AOR (95% CI)	p
Alcohol	**2.16 (1.75, 2.66)**	**<0.001**	**1.98 (1.53, 2.57)**	**<0.001**
Nicotine	**3.91 (2.65, 5.77)**	**<0.001**	**3.37 (2.16, 5.26)**	**<0.001**
Cannabis	**6.06 (3.54, 10.38)**	**<0.001**	**4.65 (2.46, 8.77)**	**<0.001**
Any substance use	**2.31 (1.89, 2.82)**	**<0.001**	**2.09 (1.64, 2.66)**	**<0.001**

Bold indicates p < 0.05. β, coefficient from linear regression model; AOR, adjusted odds ratio from logistic regression model. Models represent the abbreviated output from the logistic and linear regression models. Adjusted models include adjustment for age, sex, race/ethnicity, household income, parent education, study site, and the respective mental health, suicidal behavior, or substance use experimentation measure at Year 2. Sample weights from the Adolescent Brain Cognitive Development Study were applied based on the American Community Survey from the US Census. CBCL, Child Behavior Checklist.

We conducted several sensitivity analyses for the associations between cyberbullying victimization and mental health and substance use experimentation. When stratified by sex, we found that the prospective associations between cyberbullying victimization and somatic and attention/deficit problems were significant only in male adolescents, while depressive problems and suicidal behaviours were only significant in female adolescents (Supplementary Table S2). After adjusting for total screen time in Year 2 in addition to other covariates, findings were mostly similar, although the association with depressive problems was attenuated and no longer statistically significant (Supplementary Table S3). In the model excluding participants with any substance use experimentation at Year 2 (representing new substance use experimentation), cyberbullying victimization remained prospectively associated with new substance use experimentation outcomes, and odds ratios were relatively similar in models 2 and 3 (Supplementary Table S4).

Supplementary Table S5 shows associations between cyberbullying perpetration and mental health and substance use experimentation outcomes. There were no significant associations between cyberbullying perpetration and mental health outcomes in adjusted models. However, cyberbullying perpetration was associated with higher odds of suicidal behaviours and substance use experimentation one year later (3.48 [1.30, 9.31] and 3.35 [1.66, 6.76], respectively).

## Discussion

In a demographically diverse nationwide longitudinal cohort of 9799 11- to 12-year-old early adolescents in the US, the current study found that cyberbullying victimization was prospectively associated with substance use experimentation and higher depressive, somatic, and attention-deficit/hyperactivity problems and suicidal behaviours at one-year follow-up, after adjusting for sociodemographic covariates. In the linear regression models, depressive, somatic, and attention-deficit/hyperactivity problems had small effect sizes. In the logistic regression models, alcohol and any substance use experimentation had medium effect sizes, while nicotine use, cannabis use, and suicidal behaviours had large effect sizes. These results remained significant even after adjusting for mental health scores, suicidal behaviours, or substance use experimentation at baseline.

### Depressive symptoms

Our finding that cyberbullying victimization is prospectively associated with higher depressive symptoms one year later is consistent with prior literature. A meta-analysis of 57 cross-sectional and longitudinal studies found a positive, moderate effect of cyberbullying victimization on depression among adolescents^58^; however, 40 of the 57 included studies were cross-sectional. Of the longitudinal studies included, the one with the largest sample included 4043 early adolescents (average age = 9.93 years, *SD* = 0.71) in China who were surveyed every 6 months at five time points; cyberbullying victimization was found to be significantly associated with subsequent depressive symptoms.^59^ Our results, drawn from a demographically diverse, large longitudinal cohort of early adolescents in the US, extend the literature by providing evidence that cyberbullying victimization predicts subsequent depressive symptoms one year later. Supporting this finding that the effects of cyberbullying victimization can be enduring, one longitudinal study showed that baseline cyberbullying victimization predicted subsequent depressive symptoms at two- and five-year follow-ups among ninth graders in China.^60^

The links between cyberbullying victimization and subsequent depressive symptoms may be partially explained by Pearlin's stress process model.^61^ The stress process model posits that cyberbullying victimization as the initial stressor could trigger a series of secondary stressors, such as decreased physical activity, dietary quality, sleep quality, academic achievement, and self-esteem, which can lead to and exacerbate depressive symptoms.^60^ It has been shown that lower physical activity, dietary quality, sleep quality, and academic achievement mediated the longitudinal relationship between initial cyberbullying victimization and subsequent depressive symptoms in a sample of adolescents.^60^ Cyberbullying could also contribute to feelings of isolation and loneliness, which could increase depressive symptoms. Finally, cyberbullying could be linked with problematic screen use, which can include elements of addiction such as continued use despite negative consequences, loss of control of the amount of time spent on screens, feelings of distress when denied access to screens, and impairment in school performance.^62^ Problematic screen use is also associated with depression and poorer mental health.^62^

### Somatic problems

Our finding of a prospective association between early adolescent cyberbullying victimization and somatic problems (i.e., an extreme focus on physical symptoms, such as pain or fatigue, that causes emotional distress and impaired functioning) confirms and builds upon prior, mostly cross-sectional research. Two large-scale cross-sectional studies, one among 24,099 students across Italy aged 13 years,^63^ and the other among 188,003 students across 38 European countries aged 10–16 years,^64^ found cyberbullying victimization to be associated with more self-reported somatic complaints. Longitudinal studies that examined the relationship between cyberbullying victimization and subsequent somatic symptoms are scarce.^65^^,^^66^ In a study of 1024 students aged 12–18 years from five high schools in southern Spain, baseline cyberbullying victimization predicted self-reported somatic complaints four months later.^66^ This study did not account for somatic complaints at baseline, and we extended its four-month follow-up to an entire year. Mechanisms underlying the effects of cyberbullying victimization on subsequent increases in somatic problems may overlap with those that have been suggested for in-person peer victimization.^67^ For instance, physical symptoms may result from a suppression of immune system functioning following the release of stress hormones in response to a stressful event.^68^ Physical symptoms may also be a more “socially acceptable” alternative expression or manifestation of the psychological symptoms associated with adjustment difficulties, and physical symptoms may elicit more positive attention, sympathy, or support from adults and peers.^68^

### Attention problems

Our finding of a prospective association between early adolescent cyberbullying victimization and ADHD symptoms fills a gap in the literature. While some literature indicates that children and adolescents with ADHD are at increased risk for cyberbullying victimization,^69^ very few studies have examined the associations between baseline cyberbullying victimization and subsequent ADHD-like symptoms. However, cyberbullying victimization could be a traumatic experience that could lead to an inability to concentrate, being easily distracted, or failing to complete tasks, which could present as symptoms of inattention.^44^

### Suicidal behaviours

Our finding that early adolescent cyberbullying victimization and perpetration are prospectively associated with higher odds of suicidal behaviours expands on the few prior longitudinal studies examining the relationship between cyberbullying and suicidal behaviours, which have had mixed findings. A recent systematic review identified four longitudinal studies that measured associations between cyberbullying victimization and subsequent suicidal behaviours among children and young people.^70^ Out of those four studies,^32^^,^^71^, ^72^, ^73^ two studies found a significant association between baseline cyberbullying victimization and subsequent suicidal ideation: at four-month follow-up among a sample of 835 adolescents aged 12–18 in southern Spain,^73^ and at one-year follow-up among a predominantly male sample of 2150 at-risk youth aged 13–20 in Israel.^71^ Compared to the current study, all four studies identified in the systematic review included smaller, less demographically diverse samples, limiting the generalizability of their findings. Not included in the systematic review is a recent study in India with a larger cohort of adolescents aged 10–19 years (*n* = 4428 males, 11,864 females), which found a significant association between cyberbullying victimization and suicidal ideation cross-sectionally (AOR 2.50, 95% CI 2.09–2.98, p < 0.01), but not three years later (AOR 1.08, 95% CI 0.83–1.40).^74^ Our study adds to the body of evidence of the longitudinal effects of cyberbullying victimization on suicidality.

The interpersonal theory of suicide could explain potential links between cyberbullying victimization and suicidal behaviours.^75^ The theory posits that thwarted belongingness (i.e., experiencing a sense of isolation due to the lack of mutually caring connections with others) and perceived burdensomeness (i.e., the perception that one is a burden on those around them) could increase the risk for suicidal behaviors.^75^ Cyberbullying victimization could lead to feelings of isolation and perceived burdensomeness, which could, in turn, lead to suicidal thoughts and behaviours. Suicidal behaviours could also be a means of coping with cyberbullying victimization; for instance, death would mean that experiencing cyberbullying victimization would stop.

Fewer studies have examined the longitudinal associations between cyberbullying perpetration and suicidal behaviours. One study with a predominantly male sample of at-risk adolescents (*n* = 2150) in Israel found those involved in cyberbullying perpetration to be twice as likely than those uninvolved in cyberbullying to report suicidal ideation (OR = 2.04) or suicide attempts (OR = 2.64) in the subsequent year; these associations remained significant even after adjusting for baseline depression, hostility, and traditional bullying perpetration.^71^ To our knowledge, this study by Benatov et al. is the first to examine longitudinal associations between cyberbullying perpetration and suicidal thoughts and behaviour. Additionally, it should be noted that suicide attempts comprise a small portion of self-harm episodes. A study of 13- to 14-year-old students (*n* = 1195) in Southwest England found no association between perpetrator-only involvement in cyberbullying and self-reported self-harm (intentional self-harm, irrespective of whether suicidal intent was present) one year later, after adjusting for baseline symptoms of anxiety and depression.^76^ The higher prospective risk of suicidal behaviours among cyberbullying perpetrators identified in our unadjusted model may be partly explained by the possibility that early adolescents who reported experiences of cyberbullying perpetration also reported experiences of cyberbullying victimization. Bullying perpetration and victimization seem to co-occur more frequently online than offline, with an increased likelihood to perpetrate cyberbullying as a form of retaliation or protection among cyberbullying victims who experienced greater or more frequent aggression.^77^^,^^78^

Although not investigated in our analyses, the ideation-to-action theoretical framework of suicide may partially explain the prospective risk for suicidal behaviours among cyberbullying perpetrators, such that repeated experiences of cyberbullying perpetration among other forms of aggression may lead to habituation to destructive acts towards others and self, decreased fear of death, and increasing capability for suicide.^79^ Drawing from prior models, such as the General Aggression Model, diathesis-stress models, and emotional dysregulation theories, other potential intrapersonal and interpersonal factors that may link together cyberbullying perpetration and suicidal behaviours and warrant further research include impulsivity, anger towards self and or others, and social connectedness.^71^^,^^80^, ^81^, ^82^ Thus, further longitudinal research is indicated to more conclusively disentangle the unique effects of cyberbullying involvement (as a perpetrator, victim, or both) on suicidal thoughts and behaviours, and its underlying mechanisms.

### Substance use experimentation

The finding of prospective associations between early adolescent cyberbullying (both victimization and perpetration) and alcohol, nicotine, and cannabis use experimentation builds upon prior mostly cross-sectional studies, with the literature focusing more on cyberbullying victims and less on cyberbullying perpetrators.^39^ The first study to report the temporal associations between cyberbullying victimization and substance use (alcohol, nicotine, cannabis, cocaine, speed, LSD, ecstasy, hashish, and others) examined these self-reported variables six months apart in adolescents aged 13–17 years (*n* = 845) across ten high schools in a Spanish province.^83^ While substance use at baseline predicted increased cyberbullying victimization six months later, the path from baseline cyberbullying victimization to substance use six months later was not significant. Consistently, a study with a younger cohort of adolescents (*n* = 1542; mean age = 12.71 years, *SD* = 0.98) from three middle schools in the southeastern United States found that cyberbullying victimization did not predict subsequent substance use (alcohol, cigarettes, cigars, marijuana, and inhalants) three months later.^84^

While both of these longitudinal studies featured shorter intervals (three or six months), two longitudinal studies with an interval period of at least 12 months found adolescents who were victims of cyberbullying to be at increased risk of subsequent substance use at follow-up.^85^^,^^86^ One of these studies, with a cohort of 2768 tenth graders (mean age = 15.52 years, *SD* = 0.52) from ten Californian high schools, found that being a witness and victim of cyberbullying at baseline was associated with increased odds of substance use (past six months alcohol, cigarettes, hookah, cigars, e-cigarettes, and marijuana use) at 12-month follow-up, relative to having no cyberbullying involvement at baseline.^85^ Likewise, a three-year longitudinal study of adolescents aged 13–15 years (*n* = 867) from the midwestern United States found that baseline cyberbullying victimization correlated with greater substance use (alcohol, cigarettes, cocaine, hallucinogens, heroin, inhalants, and prescription drugs) one year and two years later.^86^ These previous studies assess substance use quantity and frequency following cyberbullying victimization, but they do not specify to what extent these rates include the initiation of substance use. Our study examines the prospective impact of cyberbullying victimization on substance use experimentation and, in a separate analysis, initiation of substance use experimentation, highlighting the importance of monitoring youth prior to the onset of significant substance use and identifying predictors of substance use experimentation for early substance use disorder prevention. Our finding that baseline cyberbullying victimization is associated with substance use experimentation at 12-month follow-up is consistent with these prior longitudinal studies with a longer interval period between baseline and follow-up, suggesting that substance use experimentation may occur in response to chronic emotional distress, or perhaps due to an association with peers who make harmful use of substances.^87^^,^^88^ This emphasizes the importance of capturing the long- and short-term effects of cyberbullying victimization, particularly among a larger, more demographically diverse cohort, to pinpoint whether substance use experimentation occurs as an acute response to cyberbullying or develops over time.^87^

Although the theoretical basis underlying the relationship between cyberbullying victimization and substance use is not yet well established, substance use may represent an avoidance coping strategy or self-medication mechanism in response to strong negative affect and emotional or psychological distress associated with online victimization or perpetration and peer rejection,^89^^,^^90^ as has been demonstrated and discussed in greater detail for other forms of bullying.^91^^,^^92^ Additionally, according to Agnew's general strain theory,^93^ the negative emotions (e.g., anger, frustration, and fear of future victimization) associated with the social strain of (cyber)bullying can motivate victims to engage in more externalizing and/or risky and illicit behaviours, such as experimentation of substance use.^94^

In line with our study findings, a meta-analysis of 28 studies on prospective associations between traditional and cyberbullying perpetration and later substance use found children and adolescents who bully to have a higher risk of drug, alcohol, and nicotine use later in life, relative to their nonbullying peers.^95^ Although bullying perpetration appears to be a risk factor for subsequent substance use, further exploration of possible individual-level and environmental mechanisms (e.g., impulsivity, emotion regulation difficulties, bullying and substance use as forms of externalizing maladjustment and/or methods for maintaining or achieving higher peer social status, association with delinquent peers, and family/home environment and parental substance use) that underlie the pathway from cyberbullying perpetration to substance use would help provide a basis for preventive interventions.^95^

### Limitations and strengths

The limitations of this study should be noted. Limitations include the observational nature of the study, leading to susceptibility to residual or unmeasured confounders despite adjustment for potential confounders. For instance, screen time could be a confounder or a partial mediator of the associations between cyberbullying and mental health outcomes. In sensitivity analyses adjusting for Year 2 screen time, findings were similar, but some were slightly attenuated. Screen time data that temporally precedes the lifetime cyberbullying measure in the ABCD Study were not available so we were not able to fully evaluate screen time as a confounder. Furthermore, the screen time was self-reported rather than objectively measured, which may limit accuracy, as participants tend to underreport smartphone screen time.^96^ Additionally, the duration of screen usage is not necessarily a reliable proxy for harmful usage,^62^ so our supplementary results that were adjusted for screen time should be interpreted with caution. Self-reported cyberbullying, mental health, and substance use experimentation measures could be subject to reporting, recall, and social desirability bias. The assessment of cyberbullying victimization used a single item, which has not been formally validated, and psychometric properties are unknown, although it was based on the Cyberbullying Scale.^42^^,^^43^ Future research could use a validated cyberbullying scale with multiple items, as well as validated measures of problematic screen or smartphone use,^62^^,^^97^ to better delineate the complex interrelationships among cyberbullying, problematic screen use, and mental health. Selection bias is also possible, although there were no significant differences in demographics among participants included versus excluded due to missing data. The low prevalence of some exposures (e.g., cyberbullying perpetration) and outcomes (e.g., cannabis) did not allow for subgroup analyses, but this could be an area of future research. Strengths include a prospective analysis of a large, demographically diverse, contemporary sample of early adolescents in the US. The sample has good generalizability to the US adolescent population given the robust sampling strategy, diversity by race, ethnicity, and socioeconomic status, and application of sampling weights based on the American Community Survey of the US Census.

### Conclusion

Our findings of prospective mental health and substance use experimentation associations with prior cyberbullying victimization have clinical, policy, and public health implications, particularly to inform the adaptation and implementation of digital technology guidance for adolescents and their parents. Pediatricians and mental health care professionals may consider assessing for cyberbullying and provide mental health support and anticipatory guidance for adolescents who report cyberbullying victimization.^98^ Digital literacy curricula in schools could also teach early adolescents to develop protective strategies to prevent cyberbullying victimization and discourage engagement in cyberbullying perpetration.^99^ Both the US Surgeon General and the American Psychological Association^100^ released advisories on adolescent social media in 2023, advocating for parents to teach children about responsible online behaviours and minimize their children's exposure to cyberbullying. Future research could provide further guidance to mitigate potential mental health consequences associated with cyberbullying as well as guidance to prevent early adolescent cyberbullying, such as identifying problematic screen use patterns most associated with cyberbullying.^101^ Investigating the extent to which cyberbullying-specific effects contribute to mental health consequences above and beyond in-person bullying is also an important area of future research.

## Contributors

J.M.N.: Writing—review & editing, Writing—original draft, Formal analysis, Data curation, Conceptualization, Supervision. He verified the data, had access to raw data, and had final responsibility for the decision to submit for publication.

J.S.: Formal analysis, Writing—original draft, Writing—review & editing, had access to raw data and verified the data.

P.B.: Formal analysis, Writing—original draft, Writing—review & editing, had access to raw data and verified the data.

A.W.L.: Writing—original draft, Writing—review & editing.

Z.S.-R.: Formal analysis, Writing—original draft, Writing—review & editing, had access to raw data and verified the data.

I.Y.S.: Formal analysis, Writing—review & editing.

A.A.A. A.-S.: Writing—original draft, Formal analysis.

C.K.H.: Writing—original draft, Formal analysis.

K.T.G.: Writing—review & editing.

A.T.: Writing—review & editing.

O.K.: Conceptualization, writing-review & editing.

J.H.: Writing—review & editing.

A.K.G.: Conceptualization, writing-review & editing.

S.B.: Conceptualization, writing-review & editing.

F.C.B.: Conceptualization, Writing—review & editing, Formal analysis, Data curation, Methodology.

## Data sharing statement

Data used in the preparation of this article were obtained from the ABCD Study (https://www.abcdstudy.org), held in the NIMH Data Archive (NDA). Researchers can access the data with a data use agreement.

## Declaration of interests

The authors have no conflicts of interest to declare.

## Appendix A Supplementary data

Supplementary Material final
