# Supplementary material for: Cyberbullying, mental health, and substance use experimentation among early adolescents: a prospective cohort study
Source: Lancet Reg Health Am. 2025 May 20;46:101002. doi: 10.1016/j.lana.2025.101002 (PMC12230417; doi:10.1016/j.lana.2025.101002)
Supplement: Supplementary Material final [file mmc1.pdf]

Cyberbullying, Mental Health, and Substance Use Experimentation Among Early Adolescents: A Prospective Cohort Study

Supplemental Material

Jason M. Nagata, M.D., Joan Shim, B.A., Priyadharshini Balasubramanian, M.P.H., Alicia W. Leong, B.A., Zacariah Smith-Russack, M.P.H., Iris Y. Shao, Ph.D., Abubakr A.A. Al-Shoaibi, Ph.D., Christiane K. Helmer, M.P.H., Kyle T. Ganson, Ph.D., Alexander Testa, Ph.D., Orsolya Kiss, Ph.D., Jinbo He, Ph.D., Allison K. Groves, Ph.D., Sarah Baird, Ph.D., Fiona C. Baker, Ph.D.

Table of contents:

1. Supplementary figure 1
2. Supplementary table 1
3. Supplementary table 2
4. Supplementary table 3
5. Supplementary table 4
6. Supplementary table 5

Supplementary figure 1. Flow diagram of inclusion criteria for participants in our study

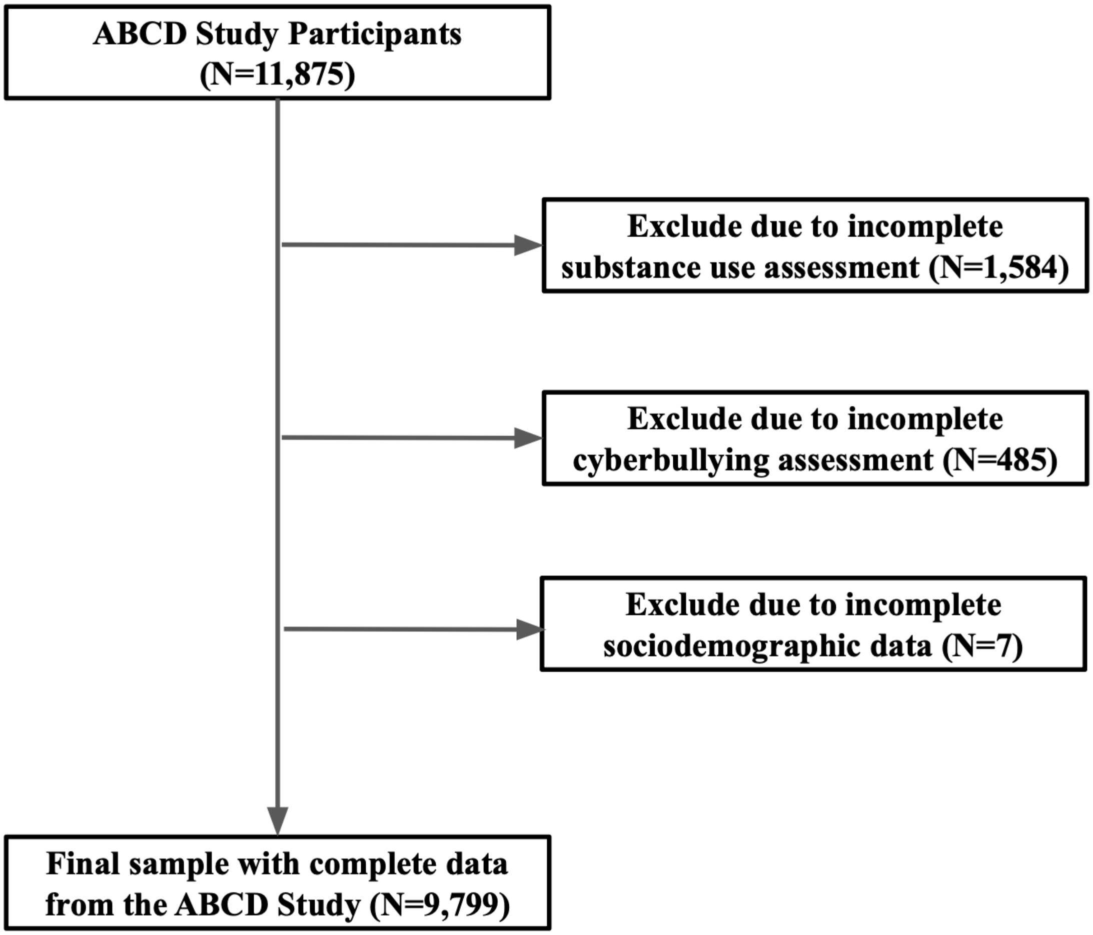

Supplementary table 1. Comparison of sociodemographic characteristics of Adolescent Brain Cognitive Development (ABCD) Study participants included versus excluded

	Included (N = 9,799)	Excluded (N = 2,076)	
**Sociodemographic characteristics**	**Mean (SD) / %**	**Mean (SD) / %**	**p[a]**
Age (years)	12.02 (0.66)	11.96 (0.75)	0.559
Sex (%)			0.836
Female	48.4%	49.8%	
Male	51.6%	50.2%	
Race/ethnicity (%)			0.517
White	54.9%	50.0%	
Latino/Hispanic	19.6%	20.8%	
Black	15.4%	23.7%	
Asian	5.5%	3.9%	
Native American	3.2%	1.6%	
Other	1.4%	0.0%	
Household income (%)			0.229
$24,999 or less	15.8%	21.7%	
$25,000 to $49,999	19.9%	27.6%	
$50,000 to $74,999	18.0%	17.1%	
$75,000 to $99,999	14.2%	10.1%	
$100,000 to $199,999	24.3%	23.1%	
$200,000 and greater	7.8%	0.4%	
Parent's highest education			0.557
College education or more	82.5%	79.3%	
High school education or less	17.5%	20.7%	

ABCD Study sampling weights were applied based on the American Community Survey from the US Census.

[a] Independent samples t-test for continuous variables, chi-square test for categorical variables

Supplementary table 2. Sex-stratified prospective associations between lifetime victimization of cyberbullying and outcomes in mental health and substance use experimentation in the Adolescent Brain Cognitive Development (ABCD) Study (N=9,799)

	**Cyberbullying Victimization**		**Cyberbullying Victimization**	
	**Females, adjusted**		**Males, adjusted**	
Outcomes at Year 3 follow-up				
CBCL DSM-Oriented Scales	β (95% CI)	p	β (95% CI)	p
Depressive Problems	**0.83 (0.04, 1.61)**	**0.038**	0.37 (-0.48, 1.23)	0.394
Anxiety Problems	0.71 (-0.04, 1.46)	0.064	0.01 (-0.70, 0.72)	0.972
Somatic Problems	0.54 (-0.23, 1.30	0.167	**1.55 (0.70, 2.41)**	**<0.001**
Attention/Deficit	0.22 (-0.39, 0.83)	0.488	**0.85 (0.11, 1.59)**	**0.025**
Oppositional Defiant Problems	0.16 (-0.44, 0.76)	0.604	0.28 (-0.40, 0.97)	0.414
Conduct Problems	0.47 (-0.18, 1.11)	0.157	0.08 (-0.61, 0.77)	0.823
Kiddie Schedule for Affective Disorders	AOR (95% CI)	p	AOR (95% CI)	p
Suicide behaviours	**3.00 (1.88, 4.76)**	**<0.001**	1.90 (0.79, 4.59)	0.154
Substance use experimentation	AOR (95% CI)	p	AOR (95% CI)	p
Alcohol	**2.30 (1.62, 3.27)**	**<0.001**	**1.65 (1.13, 2.39)**	**0.009**
Nicotine	**4.08 (2.22, 7.51)**	**<0.001**	**2.88 (1.49, 5.56)**	**0.002**
Cannabis	**4.62 (2.06, 10.37)**	**<0.001**	**4.94 (1.88, 13.01)**	**0.001**
Any substance use	**2.38 (1.70, 3.35)**	**<0.001**	**1.80 (1.27, 2.56)**	**0.001**

Bold indicates $p<0.05$. β=coefficient from linear regression model. AOR=adjusted odds ratio from logistic regression model. Models represent the abbreviated output from the sex-stratified logistic and linear regression models. Adjusted models include adjustment for age, race/ethnicity, household income, parent education, study site, and the respective mental health, suicidal behaviour, or substance use experimentation measure at Year 2. Sampling weights from the Adolescent Brain Cognitive Development Study were applied based on the American Community Survey from the US Census. CBCL= Child Behavior Checklist

Supplementary table 3. Prospective associations between lifetime victimization of cyberbullying and outcomes in mental health and substance use experimentation in the Adolescent Brain Cognitive Development (ABCD) Study, adjusted for total screen time (N = 9,799)

	**Cyberbullying Victimization**		**Cyberbullying Victimization**	
	**Unadjusted**		**Adjusted**	
Outcomes at Year 3 follow-up				
CBCL DSM-Oriented Scales	β (95% CI)	p	β (95% CI)	p
Depressive Problems	**1.85 (1.19, 2.51)**	**<0.001**	0.51 (-0.07, 1.10)	0.084
Anxiety Problems	**1.13 (0.50, 1.76)**	**<0.001**	0.34 (-0.18, 0.87)	0.203
Somatic Problems	**1.53 (0.91, 2.14)**	**<0.001**	**0.94 (0.36, 1.51)**	**0.001**
Attention/Deficit	**1.72 (1.11, 2.33)**	**<0.001**	**0.49 (0.00, 0.98)**	**0.049**
Oppositional Defiant Problems	**1.24 (0.68, 1.80)**	**<0.001**	0.15 (-0.31, 0.60)	0.532
Conduct Problems	**1.40 (0.81, 2.00)**	**<0.001**	0.22 (-0.25, 0.69)	0.354
Kiddie Schedule for Affective Disorders	AOR (95% CI)	p	AOR (95% CI)	p
Suicide behaviours	**3.14 (2.20, 4.48)**	**<0.001**	**2.33 (1.52, 3.59)**	**<0.001**
Substance use experimentation	AOR (95% CI)	p	AOR (95% CI)	p
Alcohol	**2.16 (1.75, 2.66)**	**<0.001**	**1.92 (1.48, 2.49)**	**<0.001**
Nicotine	**3.91 (2.65, 5.77)**	**<0.001**	**3.08 (1.96, 4.83)**	**<0.001**
Cannabis	**6.06 (3.54, 10.38)**	**<0.001**	**4.11 (2.15, 7.86)**	**<0.001**
Any substance use	**2.31 (1.89, 2.82)**	**<0.001**	**1.95 (1.53, 2.50)**	**<0.001**

Bold indicates p<0.05. β=coefficient from linear regression model. AOR=adjusted odds ratio from logistic regression model. Models represent the abbreviated output from the logistic and linear regression models. Adjusted models include adjustment for age, sex, race/ethnicity, household income, parent education, total screen time, study site, and the respective mental health, suicidal behaviour, or substance use experimentation measure at Year 2. Sampling weights from the Adolescent Brain Cognitive Development Study were applied based on the American Community Survey from the US Census. CBCL= Child Behavior Checklist

Supplementary table 4. Prospective associations between lifetime victimization of cyberbullying and substance use experimentation in the Adolescent Brain Cognitive Development (ABCD) Study

	**Cyberbullying Victimization**					
	**Model 1: Unadjusted (N=9,799)**		**Model 2: Adjusted (N=9,799)**		**Model 3: Adjusted and excluding participants with substance use experimentation at Year 2 (N=8,765)**	
Outcomes at Year 3 follow-up						
Substance use experimentation	AOR (95% CI)	p	AOR (95% CI)	p	AOR (95% CI)	p
Alcohol	**2.16 (1.75, 2.66)**	**<0.001**	**1.98 (1.53, 2.57)**	**<0.001**	**1.88 (1.37, 2.58)**	**<0.001**
Nicotine	**3.91 (2.65, 5.77)**	**<0.001**	**3.37 (2.16, 5.26)**	**<0.001**	**2.84 (1.60, 5.05)**	**<0.001**
Cannabis	**6.06 (3.54, 10.38)**	**<0.001**	**4.65 (2.46, 8.77)**	**<0.001**	**3.96 (1.81, 8.67)**	**<0.001**
Any substance use	**2.31 (1.89, 2.82)**	**<0.001**	**2.09 (1.64, 2.66)**	**<0.001**	**2.08 (1.56, 2.79)**	**<0.001**

Bold indicates $p<0.05$. β=coefficient from linear regression model. AOR=adjusted odds ratio from logistic regression model. Models represent the abbreviated output from the logistic and linear regression models. Adjusted models include adjustment for age, sex, race/ethnicity, household income, parent education, study site, and the respective mental health, suicidal behaviour, or substance use experimentation measure at Year 2. Model 3 excludes participants with substance use experimentation at Year 2, thereby representing new substance use experimentation. Sampling weights from the Adolescent Brain Cognitive Development Study were applied based on the American Community Survey from the US Census. CBCL= Child Behavior Checklist

Supplementary table 5. Prospective associations between lifetime perpetration of cyberbullying and outcomes in mental health and substance use experimentation in the Adolescent Brain Cognitive Development (ABCD) Study (N = 9,799)

	**Cyberbullying Perpetration Unadjusted**		**Cyberbullying Perpetration Adjusted**	
Outcomes at Year 3 follow-up				
CBCL DSM-Oriented Scales	β (95% CI)	p	β (95% CI)	p
Depressive Problems	2.15 (-0.01, 4.31)	0.051	0.01 (-2.03, 2.05)	0.992
Anxiety Problems	0.88 (-0.86, 2.62)	0.321	0.51 (-0.96, 2.00)	0.501
Somatic Problems	1.13 (-0.41, 2.67)	0.149	0.52 (-1.40, 2.45)	0.594
Attention/Deficit	**3.28 (1.25, 5.31)**	**0.002**	1.27 (-0.49, 3.03)	0.156
Oppositional Defiant Problems	**2.45 (0.54, 4.36)**	**0.012**	0.57 (-1.06, 2.20)	0.496
Conduct Problems	**4.49 (2.19, 6.79)**	**<0.001**	1.80 (-0.04, 3.64)	0.055
Kiddie Schedule for Affective Disorders	AOR (95% CI)	p	AOR (95% CI)	p
Suicide behaviours	**3.50 (1.49, 8.23)**	**0.004**	**3.48 (1.30, 9.31)**	**0.013**
Substance use experimentation	AOR (95% CI)	p	AOR (95% CI)	p
Alcohol	**4.64 (2.76, 7.83)**	**<0.001**	**3.00 (1.44, 6.28)**	**0.003**
Nicotine	**11.20 (5.75, 21.80)**	**<0.001**	**14.77 (6.70, 32.57)**	**<0.001**
Cannabis	**18.59 (8.44, 40.95)**	**<0.001**	**11.89 (4.37, 32.33)**	**<0.001**
Any substance use	**4.83 (2.92, 7.99)**	**<0.001**	**3.35 (1.66, 6.76)**	**0.001**

Bold indicates p<0.05. β=coefficient from linear regression model. AOR=adjusted odds ratio from logistic regression model. Models represent the abbreviated output from the logistic and linear regression models. Adjusted models include adjustment for age, sex, race/ethnicity, household income, parent education, study site, and the respective mental health, suicidal behavior, or substance use experimentation measure at Year 2. Sampling weights from the Adolescent Brain Cognitive Development Study were applied based on the American Community Survey from the US Census. CBCL= Child Behavior Checklist
